# Pain and Its Therapeusis

**Published:** 1897-12

**Authors:** 


### Pain and its Therapeusis.

Dr. S. V. Clevenger, after pointing out the disadvantages of various analgesic drugs, states that lactophenin is destined to supersede largely the entire array of analgesics proper, owing to its nontoxic peculiarities and the feeling of comfort described by many physicians as following its use. It affords the best results with the least ill effects. Its range of incompatibility is less than other synthetic compounds, and it may be combined with caffein, quinin and salicylic acid. The minimum dose of 5 to 10 grains may be increased until a daily maximum of 45 grains has been reached. It is but slightly soluble in water, although acting promptly, so that it can be given dry, and be washed down with a drink of water. A dose of 15 grains usually acts as a feeble hypnotic. There are no untoward symptoms following its use, and, contrary to the experience with some synthetic drugs, the pulse becomes fuller and stronger under its use. The range of application is extensive, and the testimony of the author is in corroboration of the findings of other physicians as to its superior analgesic effects, its sefety and promptness of action.—R. W. Wilcox, M. D., in *American Journal of the Medical Sciences*, May, 1897, quoting from *Journal of the American Medical Association*, 1897, No. 5.

